# Effects of irrigation fluid temperature during flexible ureteroscopic holmium laser lithotripsy on postoperative fever and shivering: a randomized controlled trial

**DOI:** 10.1186/s12894-021-00841-4

**Published:** 2021-04-27

**Authors:** Yue He, You-Gang Feng, Jun He, Bo Liang, Ming-Dong Jiang, Jun Liu, Yong-Ming Kang, Li-Ping Ma, Qin Zhang, Qi-Jia Peng, Tao Yang, Yao Liu, Li Luo, Min Zhang

**Affiliations:** 1 Department of Urology, Suining Central Hospital, No. 127 Desheng W. Rd., Chuanshan District, Suining City, 629000 Sichuan Province People’s Republic of China; 2 Department of Urology, Institute of Urology, West China Hospital, Sichuan University, No. 37 Guoxue Alley, Wuhou District, Chengdu City, 610041 Sichuan Province People’s Republic of China; 3 Operation Room, Suining Central Hospital, No. 127 Desheng W. Rd., Chuanshan District, Suining City, 629000 Sichuan Province People’s Republic of China; 4 Department of Anesthesiology, Suining Central Hospital, No. 127 Desheng W. Rd., Chuanshan District, Suining City, 629000 Sichuan Province People’s Republic of China

**Keywords:** Irrigation fluid, Temperature, Flexible ureteroscopic holmium laser lithotripsy, Calculi, Holmium: YAG laser

## Abstract

**Background:**

Flexible ureteroscopic holmium laser lithotripsy is used to treat urinary tract calculi, but postoperative complications include shivering, fever and infection. To investigate the effects of irrigation fluid temperature on postoperative complications.

**Methods:**

This randomized controlled trial included 120 consecutive patients undergoing flexible ureteroscopic holmium laser lithotripsy at the Urology Department, Suining Central Hospital, Sichuan, China between January 2017 and July 2019. Patients were randomized 1:1:1 into three groups (17 °C, 27 °C or 37 °C). Primary outcome was fever incidence (body temperature > 37.5 °C) within 48 h after surgery. Secondary outcomes included shivering incidence during recovery from anesthesia, white blood cell count (WBC), serum procalcitonin (PCT) and incidence of suspected infection (temperature > 38.5 °C and PCT > 0.5 µg/L).

**Results:**

There were 108 patients, (17 °C group, n = 36; 27 °C group, n = 35; 37 °C group, n = 37), received flexible ureteroscopic holmium laser lithotripsy and analyzed. Age, gender distribution, body mass index, ASA grade, stone burden, preoperative creatinine, preoperative core temperature and irrigation fluid volume did not differ significantly between groups. 17 °C, 27 °C and 37 °C groups exhibited significant differences in the incidences of postoperative fever (38.9% vs. 17.1% vs. 13.5%) and shivering (22.2% vs. 5.7% vs. 2.7%) (*p* < 0.05 for all pairwise comparisons). There was no significant difference of WBC, PCT and incidence of suspected infection in 37 °C or 27 °C group compared with 17 °C group. One case each of flash pulmonary edema and bleeding occurred in 37 °C group.

**Conclusion:**

Warming the irrigation fluid can reduce the incidence of postoperative fever and shivering, but further studies are needed to determine the optimal temperature.

*Trial registration* The trial was registered at the Chinese Clinical Trials Registry and allocated as ChiCTR2000031683. The trial was registered on 07/04/2020 and this was a retrospective registration.

## Background

Calculi can develop in the urinary tract when the urine becomes supersaturated with a mineral, leading to the formation of crystals comprised of calcium oxalate, calcium phosphate, uric acid or other substances [1]. The prevalence of urinary calculi is higher in developed countries than in developing countries and has been estimated to be around 9% in the USA and 4% in China [2]. Factors associated with an increased risk of urinary calculi in adulthood include male gender, obesity and diabetes mellitus [1]. Urinary stones often present acutely with pain, infection or hematuria, necessitating treatment. A variety of medical [3] and surgical [4, 5] therapies are available for the treatment of urinary calculi, including stone fragmentation by lithotripsy [6].

The development of endoscopic technology and lithotripsy techniques for the treatment of urinary calculi has led to traditional open surgery being replaced by endoscopic procedures [7]. Ureteroscopes introduced via the urethra can reach upper urinary tract calculi in the ureter, renal pelvis and calyces, permitting holmium laser lithotripsy to be carried out as a treatment [8]. The latest recommendations of the European Association of Urology, which were published in 2019, recommend flexible ureteroscopic lithotripsy as the first-line surgical treatment option for proximal ureteral/renal calculi with a diameter < 20 mm [9]. However, postoperative complications occur in 2.5–6.7% of patients after ureteroscopic stone treatment [10–13], and fever is the most common postoperative complication of ureteroscopic holmium laser lithotripsy [10, 11, 14–18]. Therefore, there is great interest in identifying methods to reduce the incidence of fever after ureteroscopic holmium laser lithotripsy.

The use of irrigation fluid is essential during transurethral endoscopic surgery. Flexible ureteroscopic lithotripsy is often performed with continuous saline irrigation through the working channel to improve the visibility when an instrument is inserted, particularly during stone dusting or when a small venous bleed occurs. Warming the irrigation fluid has been shown to reduce the incidence of hypothermia and shivering during transurethral resection of the prostate (TURP) [19]. Furthermore, the use of isothermic irrigation fluid can help to maintain normal body temperature and avoid hypothermia during percutaneous nephrolithotripsy (PCNL) [20]. However, the effects of irrigation fluid temperature during flexible ureteroscopic holmium laser lithotripsy on body temperature and the postoperative incidences of shivering, fever, infection and other complications remain unknown.

We hypothesized that increasing the temperature of the irrigation fluid during flexible ureteroscopic holmium laser lithotripsy would reduce the incidence of shivering during recovery from anesthesia and fever during the 2-day period after the procedure. Therefore, the aim of this randomized controlled trial was to investigate the effects of irrigation fluid temperature during flexible ureteroscopic holmium laser lithotripsy on the incidence of short-term postoperative complications including fever and shivering.

## Methods

### Study design

This was a randomized controlled trial conducted at the Department of Urology, Suining Central Hospital, Sichuan, China between January 2017 and July 2019. The Ethics Committee of Suining Central Hospital, Sichuan Province, P.R. China approved the study. All patients read and understood written information describing the study aims and protocol, and all patients signed consent forms agreeing to participate in this clinical research.

### Patients

Participants were enrolled using the following inclusion criteria: (1) age 18–70 years; (2) American Society of Anesthesiologists (ASA) grade I or II [21]; (3) diagnosis of unilateral proximal ureteral calculi and/or renal calculi made by computed tomography (CT); (4) diameters of all calculi were < 20 mm; and (5) total stone burden was < 360 mm2. The exclusion criteria were as follows: (1) thyroid disease or dysautonomia; (2) bilateral upper urinary tract stones scheduled to be treated simultaneously during surgery; (3) preoperative fever; (4) urinary tract infection when included in the trial; (5) hydronephrosis > 3 cm on ultrasound; (6) ureteral stenosis.

### Randomization and blinding

Patients were randomized 1:1:1 to 37 °C group, 27 °C group or 17 °C group. The random sequence and grouping sequence were generated using the random function in Excel (Microsoft Corp., Redmond, WA, USA), and the generated random sequence was put into sequentially coded, sealed and opaque envelopes. The envelopes were kept by an investigator (TY) who did not participate in the recruitment of patients, intervention or outcome evaluation. The sealed envelope was brought into the operating room with the patient and opened by the anesthesiologist and nurse who were in charge of setting the temperature of the irrigation fluid during surgery.

### Intervention

One week before the procedure, a ureteral stent was placed routinely to dilate the ureter. The irrigation fluid used during surgery was sterile physiological saline. The surgical nurse (MLP, ZQ, PQJ), who was responsible for setting the temperature of the irrigation fluid during the operation, placed the irrigation fluid into a medical incubator with a pre-set temperature (37 °C, 27 °C or 17 °C) 6 h before the procedure. The temperature of the irrigation fluid was 37 °C for patients in 37 °C group, 27 °C for patients in 27 °C group, and 17 °C for patients in 17 °C group. Intravenous fluids were given at room temperature to patients in all three groups.

The procedure was carried out under general anesthesia with the patient in the lithotomy position and covered with a single layer of sterile surgical drape. The operating room temperature was adjusted to 24 °C. The intraoperative core (tympanic) temperature was recorded by the anesthesiologist (ZM, LL) every 10 min until the end of the operation. No heating device was used until the core temperature dropped below 34 °C. All procedures were performed by the same surgical team, which was led by a chief surgeon (HJ, HY, FYG) with previous experience of more than 50 ureteroscopic holmium laser lithotripsy procedures.

The Olympus URF-V electronic flexible ureteroscope were used in the operation. For irrigation, a 3-L bag of sterile 0.9% NaCl solution was suspended 70 cm above the kidney. One end of the infusion tube was connected to the fluid bag, and the other end was connected to the water inlet switch of the flexible ureteroscope. The chief surgeon was able to use the water inlet switch to control the fluid inlet flow. In each case, ureteral access sheath (internal and external size: Fr12 and Fr14) was used. The laser fiber was 200 µm, and the basket was COOK (NGE-017115-UDH-MB). Primary technique was dusting to break up the stones. The laser parameters: pulse energy (1 J), frequency (20 Hz). If the diameter is greater than 3 mm, basket was used. A ureteral stent was placed routinely after surgery, about for one month after treatment. Following completion of the operation, the patient was transferred from the operating room to the post-anesthesia care unit (PACU) and returned to the general ward once fully awake. Routine blood tests, measurement of serum procalcitonin (PCT) and renal function tests were performed 24 h after surgery, and body temperature was recorded. The urinary catheter was removed 24 h after the operation, and the patient was discharged 48 h after surgery in the absence of serious complications.

### Clinical data and outcomes

Each patient’s age, gender, body mass index (BMI), preoperative serum creatinine level, ASA grade, stone location and stone burden were recorded before the intervention. Postoperative parameters including core body temperature at the end of surgery, occurrence of postoperative shivering, maximum body temperature during the 48 h after the operation, white blood cell (WBC) count, serum PCT level, serum creatinine level and duration of hospitalization were also collected for analysis.

Stone burden was defined as the two-dimensional area determined by multiplying the longest diameter by the perpendicular diameter of the stone [22]. In cases of multiple stones, the total (cumulative) stone burden was calculated as the sum of the burden of each stone. Postoperative fever was defined as any temperature reading above 37.5 °C. The combination of body temperature > 38.5 °C and PCT > 0.5 µg/L was considered as suspected infection, necessitating further investigation and the administration of antibiotics and/or delay of discharge, as necessary.

The primary outcome of this study was the incidence of fever (body temperature > 37.5 °C within 48 h after the operation). The secondary outcomes were the incidence of shivering during recovery from anesthesia, WBC count, serum PCT level, incidence of suspected infection and length of hospital stay. Complications were classified according to the modified Clavien-Dindo grading system [23].

### Sample size

Calculation of sample size was done on the basis of the formula ${\text{n}} = \frac{1641.6\lambda }{{(\sin^{ - 1} \sqrt {Pmax} - \sin^{ - 1} \sqrt {Pmin} )^{2} }}$, assuming a statistical power (β) of 90% and an α error of 5%. According to the literature, the incidence of fever following ureteroscopy is less than 5%. Using estimated fever rates of Pmin = 5% and Pmax = 35%, the calculated minimum sample size for each group was n = 38. Assuming a rate of exclusion or loss to follow-up of 5%, the study was designed to include 40 patients in each group.

### Statistical analysis

The data were analyzed using SPSS 19.0 (IBM Corp., Armonk, NY, USA). Normally-distributed continuous data are described as mean ± standard deviation and were compared between groups using one-way analysis of variance (ANOVA) and the Bonferroni post-hoc test. Non-normally distributed data are described as median (range) and were compared between groups using the Kruskal Wallis H test. Dunnett’s test was used to perform multivariate comparisons between 37 °C group and 27 °C group, 17 °C group, respectively. Qualitative data were analyzed using Pearson’s chi squared test or Fisher’s exact test with Bonferroni correction for multiple comparisons. A *p*-value < 0.05 was accepted as statistically significant.

## Results

### Enrolment of the study participants

**Fig. 1:**
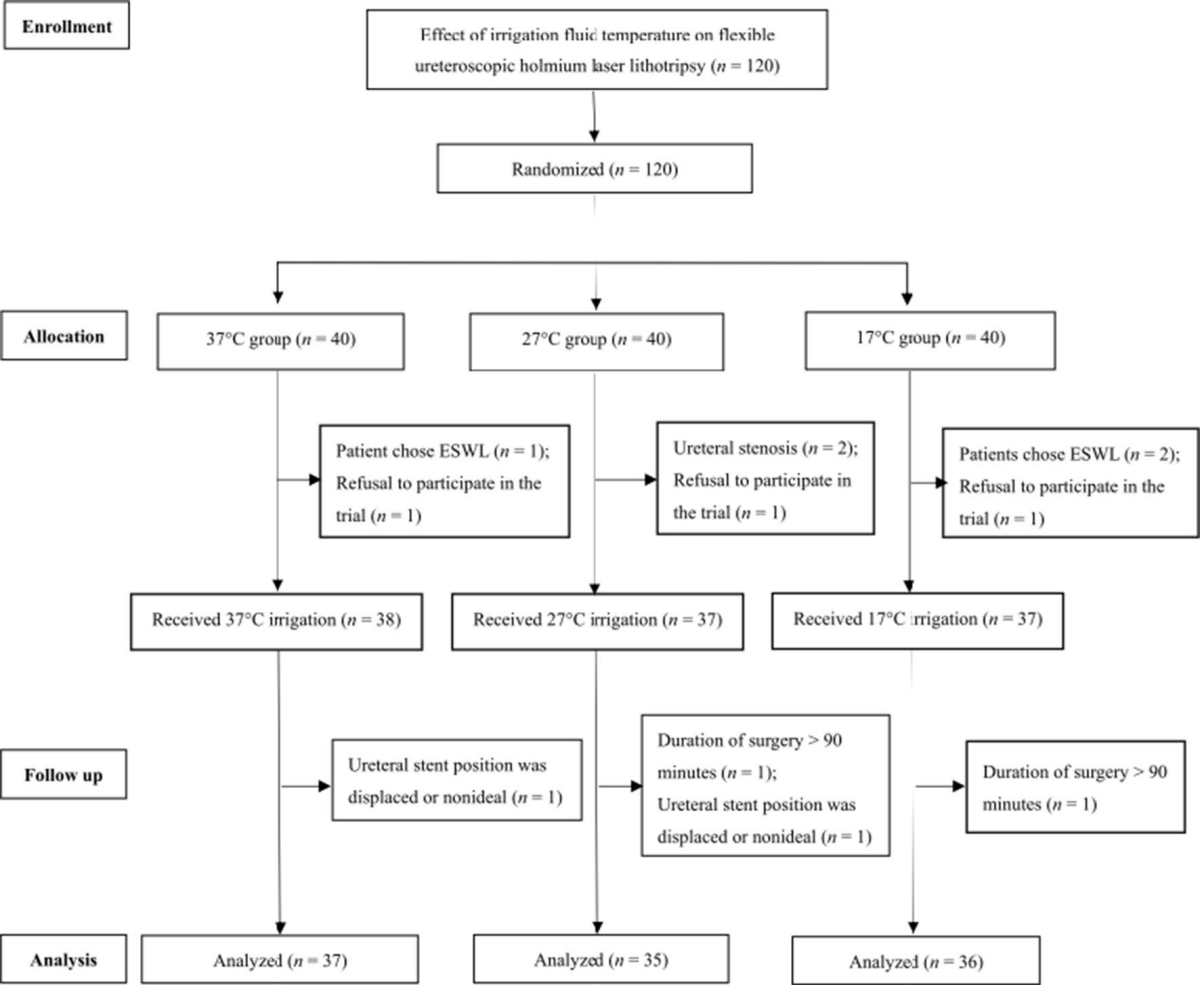
Flow-diagram of the trial

Among 120 patients initially randomized to the three study groups, a total of 12 patients were subsequently excluded from the analysis due to refusal to participate in the trial (n = 3), refusal of flexible ureteroscopic holmium laser lithotripsy in favor of extracorporeal shock wave lithotripsy or PCNL (n = 3), duration of surgery > 90 min (n = 2), ureteral stenosis (n = 2) or nonideal positioning or displacement of the ureteral stent (n = 2). Therefore, the final analysis included 37 patients in 37 °C group, 35 patients in 27 °C group and 36 patients in 17 °C group (Fig. 1).

### Preoperative clinical characteristics

There were no significant differences between the three groups in age, sex distribution, BMI, stone location, stone burden, ASA grade or preoperative serum creatinine level (Table 1).

**Table 1:** Preoperative clinical characteristics of the study participants

	37 °C (*n* = 37)	27 °C (*n* = 35)	17 °C (*n* = 36)	*p*
Male, n (%)	20 (54.1%)	16 (45.7%)	19 (52.8%)	0.750
Female, n (%)	17 (45.9%)	19 (54.3%)	17 (47.2%)	
Age (years)	42.8 ± 12.3	44.9 ± 13.1	44.3 ± 11.7	0.758
Body mass index (kg/m^2^)	24.11 ± 2.88	24.17 ± 2.69	24.26 ± 3.02	0.973
Preoperative creatinine (µ mol/L)	93.0 ± 19.0	92.6 ± 20.0	94.1 ± 18.6	0.942
*ASA grade, n (%)*				
I	23 (62.2%)	21 (60.0%)	20 (55.6%)	0.843
II	14 (37.8%)	14 (40.0%)	16 (44.4%)	
*Stone location, n (%)*				
Kidney	9 (24.3%)	7 (20.0%)	11 (30.6%)	0.830
Ureter	9 (24.3%)	8 (22.9%)	6 (16.7%)	
Kidney and ureter	19 (51.4%)	20 (57.1%)	19 (52.7%)	
Stone burden (mm^2^)	109 (40–266)	123 (48–304)	140 (45–266)	0.670

Data are presented as n (%), mean ± standard deviation or median (range). ASA, American Society of Anesthesiologists

### Intraoperative characteristics

**Fig. 2:**
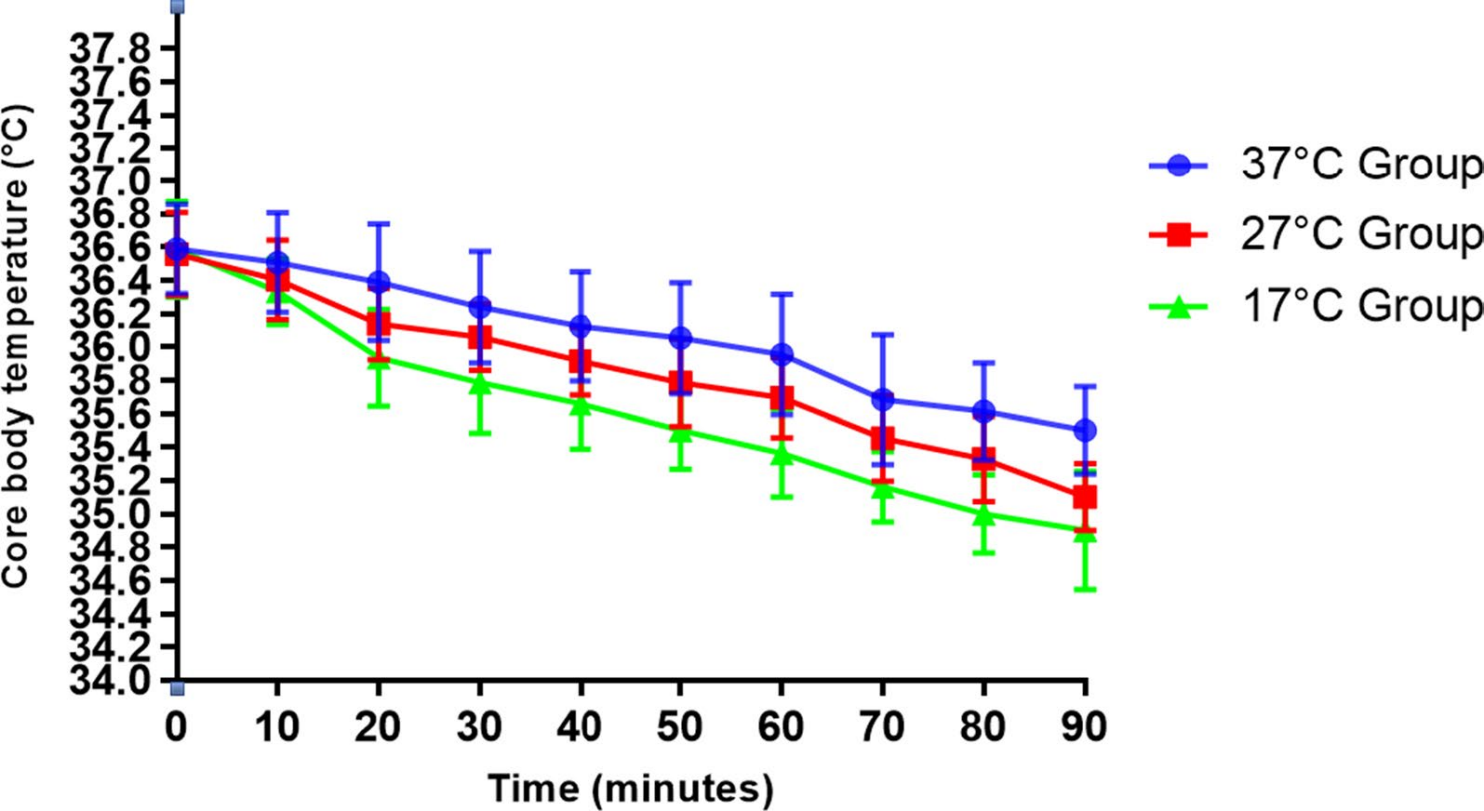
Intraoperative core body temperature for patients in the three groups

The volume of intraoperative irrigation fluid used and operation time did not differ significantly between groups (Table 2). Core body temperature declined during surgery in all three groups but tended to decrease most rapidly in 17 °C group and most slowly in 37 °C group (Fig. 2). Although preoperative core body temperature did not differ significantly between groups, core body temperature at the end of surgery was significantly lower in 17 °C group than in 27 °C group or 37 °C group (*p* < 0.05; Table 2).

**Table 2:** Perioperative and postoperative characteristics

	37 °C (*n* = 37)	27 °C (*n* = 35)	17 °C (*n* = 36)	*p*
Irrigation fluid volume (L)	1.58 ± 0.47	1.69 ± 0.51	1.77 ± 0.56	0.274
Operative time (min)	59.5 ± 14.2	60.7 ± 15.3	63.1 ± 14.6	0.573
Core temperature at start of surgery (°C)	36.59 ± 0.27	36.56 ± 0.25	36.59 ± 0.29	0.858
Core temperature at end of surgery (°C)	35.91 ± 0.36*	35.56 ± 0.32*	35.24 ± 0.36	< 0.001
Shivering during resuscitation from anesthesia	1 (2.7%)*^Δ^	2 (5.7%)*	8 (22.2%)	0.023
Postoperative creatinine (µmol/L)	96.0 ± 19.7	94.0 ± 20.6	97.0 ± 19.5	0.815
Maximum T (°C)	37.2 (36.6–39.6)*^Δ^	37.4 (36.9–39.7)	37.45 (36.9–40.0)	0.028
White blood cell count (×10^9^/L)	9.20 ± 2.58	9.43 ± 2.16	10.20 ± 2.43	0.175
Serum procalcitonin (ng/mL)	0.15 (0.03–0.60)	0.15 (0.05–0.95)	0.15 (0.05–0.97)	0.625
Fever	5 (13.5%)*^Δ^	6 (17.1%)*	14 (38.9%)	0.022
Suspected infection^a^	3 (8.1%)	2 (5.7%)	5 (13.9%)	0.505
Median hospital stays (days)	3 (3–7)	3 (3–7)	3 (3–7)	0.060

Data are presented as n (%), mean ± standard deviation or median (range). T, temperature

^*^p < 0.05 versus 17 °C group; Δ *p* < 0.05 versus 27 °C group

^a^Suspected infection was defined as core body temperature > 38.5 °C and serum procalcitonin level > 0.5 µg/L

### Primary and secondary outcomes

The incidence of postoperative fever (core body temperature > 37.5 °C during the 48 h after surgery) differed significantly between 37 °C group (5/37, 13.5%), 27 °C group (6/35, 17.1%) and 17 °C group (14/36, 38.9%) (*p* < 0.05 for all pairwise comparisons; Table 2). Similarly, the incidence of postoperative shivering differed significantly between 37 °C group (1/37, 2.7%), 27 °C group (2/35, 5.7%) and 17 °C group (8/36, 22.2%) (*p* < 0.05 for all pairwise comparisons; Table 2).

Postoperatively there were no significant differences between groups in the incidence of suspected infection (core body temperature > 38.5 °C and PCT > 0.5 µg/L), WBC count, PCT level or creatinine level, duration of hospital stays (Table 2).

### Other complications

There was one case of flash pulmonary edema (the patient recovered and was discharged after medical therapy) and one case of postoperative hemorrhage (which stopped 3 days after placement of a three-cavity catheter for bladder irrigation) in 37 °C group. There was one case of urinary retention (the catheter was successfully removed after 1 week) in 37 °C group and one case of ureteral abrasion (the ureteral stent was removed two months later) in 17 °C group. There were no adverse events above grade III (Clavien-Dindo grading system) in this study.

## Discussion

Important findings of the present study were that the incidences of postoperative fever and shivering decreased significantly as the temperature of the irrigation fluid was increased from 17 to 37 °C. Postoperative WBC count, serum PCT level and incidence of suspected infection were comparable between groups. Although there was one case of flash pulmonary edema and one case of bleeding in 37 °C group, both complications resolved after therapy. Our novel findings suggest that isothermic irrigation during flexible ureteroscopic holmium laser lithotripsy can reduce the incidence of postoperative fever and shivering. However, further studies are required to establish the optimal temperature for the irrigation fluid.

There are many potential causes of fever after ureteroscopic holmium laser lithotripsy, including ureteral obstruction by stone fragments, urinary tract infection, intraoperative backflow and extravasation of urine due to prolonged high-pressure irrigation, intraoperative and postoperative bleeding, and postoperative backflow of urine in the bladder and ureter due to poor drainage via the catheter [24]. Furthermore, hypothermia and shivering can occur due to the large amounts of intravenous and irrigation fluids administered during surgery and anesthesia [25], particularly given that a substantial amount of irrigation fluid is absorbed by the patient [26, 27]. The present study investigated the effects of varying the temperature of the irrigation fluid on core body temperature during surgery and the incidence of postoperative fever while minimizing the influence of possible confounding factors. For example, all procedures were performed by the same experienced surgical team, irrigation fluid pressure was standardized and maintained below 40 mmHg by suspending the liquid bag at the same height, and patients with preoperative urinary tract infection or postoperative ureteral stent displacement were excluded from the analysis.

In this study, core body temperature at the end of the procedure was significantly lower in 17 °C group than in the other two groups, indicating that the use of irrigation fluid at the lower temperature (17 °C) contributed to the fall in body temperature during surgery. By contrast, core body temperature at the end of the operation did not differ significantly between 37 °C group and 27 °C group, indicating that additional heating of the irrigation fluid from 27 to 37 °C was not beneficial for maintaining body temperature during surgery. This may have been due to the energy generated by the holmium laser during the operation, which increased the local temperature and heated the irrigation fluid [28].

The main finding of this study was that the incidence of postoperative fever (core body temperature > 37.5 °C during the 48 h after surgery) was significantly lower in 27 °C group than in 17 °C group and significantly lower in 37 °C group than in both the other groups. However, there were no significant differences between the three groups in the postoperative WBC count, serum PCT level or incidence of suspected infection (core body temperature > 38.5 °C and PCT > 0.5 µg/L) [29]. The above results suggest that the differences in the incidences of fever between groups were not due to differences in the incidences of infection, further implying that the temperature of the irrigation fluid has little influence on the incidence of postoperative urinary tract infection. The use of hypothermic irrigation fluid can cause intraoperative hypothermia, and the risk of postoperative shivering is increased when core body temperature drops below 36 °C [30]. A recent meta-analysis of 28 randomized controlled trials found that the fall in body temperature during surgical treatment of benign prostatic hypertrophy and the incidence of postoperative shivering were lower for warm irrigation fluid than for room-temperature irrigation fluid [19]. Therefore, our findings are in good agreement with the results of the above meta-analysis [19] and with other studies evaluating the effects of irrigation fluid temperature on body temperature [31, 32]. Our observation that the incidence of postoperative fever increased as the irrigation fluid temperature was decreased (from 37 to 17 °C) might be explained by a compensatory reaction of the body to hypothermia at the end of surgery, with the shivering mechanism possibly contributing to this.

The incidence of postoperative fever in this study was relatively high in comparison to that reported by previous studies, ranging from 13.5 to 38.9%. This may have been due to the wide definition of fever used in this study, since the patient was classified as having fever even if only one temperature reading was above 37.5 °C during the 48 h after surgery. Ten patients (9.2%) had a postoperative body temperature higher than 38.5 °C after the operation: 5 of these patients improved after symptomatic treatment without the use of antibiotics, and the remaining 5 patients were diagnosed with urinary tract infection. The incidence of urinary tract infection in our study (4.6%) was higher than rates of 1–1.8% reported in the literature [10, 11], and we speculate that there may be three reasons for this. First, our study had a small sample size. Second, stone burden was larger in our study than in the previous studies, which may have resulted in more bacteria being released from the stone during the process of laser lithotripsy. Third, our study included more cases with renal calculi, whereas the prior studies mainly included patients with ureteral calculi.

There was one case of flash pulmonary edema in 37 °C group. It has been reported that elevating the temperature of the irrigation fluid increases the amount that is absorbed by the body [26]. Therefore, for the same irrigation pressure and duration of surgery, it would be expected that patients in 37 °C group would have absorbed a greater volume of fluid than patients in the other groups. As a result, high-temperature irrigation fluid is more likely to cause volume overload and lead to pulmonary edema than low-temperature irrigation fluid, especially in elderly patients. However, the risk of irrigation fluid absorption during flexible ureteroscopy is lower than that during TURP or PCNL because flexible ureteroscopy causes far less tissue injury than TURP and uses a smaller perfusion pressure than PCNL [27]. However, there remains a risk of increased fluid absorption for warmed irrigation fluid, which needs further study. Therefore, decisions as to whether or not to warm the irrigation fluid and the degree of warming necessitate full consideration of factors such as the predicted duration of surgery and cardiovascular risk of the patient, among others.

In addition, there was one case of postoperative bleeding in 37 °C group, but the bleeding stopped after irrigation for 24 h using a three-cavity catheter. By contrast, there were no cases of bleeding in the other two groups, raising the possibility that warming the irrigation fluid might increase the risk of bleeding. Cao et al. reported that the amount of blood lost during TURP did not differ significantly between warmed and room-temperature irrigation fluid [19]. However, Kati et al. found that the amount of blood loss during PCNL was significantly lower for patients irrigated with fluid at room temperature [20]. It will be necessary to further study the effects of irrigation fluid temperature on bleeding risk. Although the risk of bleeding is very low for most patients undergoing flexible ureteroscopy, it is higher for patients with coagulation dysfunction or on long-term oral anticoagulants.

One limitation of this study is that there was no calculation of the amount of irrigation fluid absorbed, although it should be noted that the methods used for this are not always very accurate [27]. In addition, patient comfort was not evaluated in our study.

## Conclusion

The use of heated irrigation fluid during flexible ureteroscopic lithotripsy can reduce the incidences of postoperative fever and shivering. However, further research is needed to establish the optimal temperature of the irrigation fluid while taking into account local heating effects caused by laser lithotripsy. In addition, decision-making regarding the temperature of the irrigation fluid should also take into consideration the cardiovascular risk of the individual patient and the predicted duration of surgery so as to avoid excessive absorption of irrigation fluid during the operation.

## Data Availability

Records and data pertaining to this study are in the patient’s secure medical records in Suining Central Hospital and are available from the corresponding author on reasonable request.

## Abbreviations

ASA: American Society of Anesthesiologists
CT: Computed tomography
ESWL: Extracorporeal shock wave lithotripsy
PCNL: Percutaneous nephrolithotomy
PCT: Serum procalcitonin
TURP: Transurethral resection of the prostate
WBC: White blood cell count
